# Donor financing of human resources for health, 1990–2016: an examination of trends, sources of funds, and recipients

**DOI:** 10.1186/s12992-018-0416-z

**Published:** 2018-10-17

**Authors:** Angela E Micah, Bianca S Zlavog, Catherine S Chen, Abigail Chapin, Joseph L Dieleman

**Affiliations:** Institute for Health Metrics and Evaluation, 2301 Fifth Ave., Suite 600, Seattle, WA 98121 USA

**Keywords:** Human resources for health, Development assistance, Donor funding

## Abstract

**Background:**

Skilled health professionals are a critical component of the effective delivery of lifesaving health interventions. The inadequate number of skilled health professionals in many low- and middle-income countries has been identified as a constraint to the achievement of improvements in health outcomes. In response, more international development agencies have provided funds toward broader health system initiatives and health workforce activities in particular. Nonetheless, estimates of the amount of donor funding targeting investments in human resources for health activities are few.

**Methods:**

We utilize data from the Institute for Health Metrics and Evaluation’s annual database on development assistance for health. The estimates in the database are generated using data from publicly available databases that track development assistance. To estimate development assistance for human resources for health, we use keywords to identify projects targeted toward human resource processes. We track development for human resources for health from 1990 through 2016. We categorize the types of human-resources-related projects funded and examine the availability of human resources, development assistance for human resources for health, and disease burden.

**Results:**

We find that the amount of donor funding directed toward human resources for health has increased from only $34 million in 1990 to $1.5 billion in 2016 (in 2017 US dollars). Overall, $18.5 billion in 2017 US dollars was targeted toward human resources for health between 1990 and 2016. The primary regions receiving these resources were sub-Saharan Africa and Southeast Asia, East Asia, and Oceania. The main donor countries were the United States, Canada, Australia and the United Kingdom. The main agencies through which these resources were disbursed are non-governmental organizations (NGOs), US bilateral agencies, and UN agencies.

**Conclusion:**

In 2016, less than 4% of development assistance for health could be tied to funding for human resources. Given the central role skilled health workers play in health systems, in order to make credible progress in reducing disparities in health and attaining the goal of universal health coverage for all by 2030, it may be appropriate for more resources to be mobilized in order to guarantee adequate manpower to deliver key health interventions.

**Electronic supplementary material:**

The online version of this article (10.1186/s12992-018-0416-z) contains supplementary material, which is available to authorized users.

## Background

The 2006 World Health Report estimated a global shortage of about 4.3 million health workers – doctors, midwives, nurses, and support staff [1]. The shortage of health workers is exacerbated by a global imbalance in the distribution of the available health workers. For instance, whereas sub-Saharan Africa carries 20.9% of the global burden of disease, it had 4.5% of the global health workforce in 2016 [2, 3]. The consequence of this shortage is especially acute for vulnerable populations such as pregnant women, children, and the aged, who tend to be most in need of health care.

Furthermore, in many low- and middle-income countries, the deficits in health workforce have been identified as one of the constraints to the success of health system reforms aimed at improving health outcomes [1, 4]. Especially for global health initiatives, such as the Global Fund for AIDS, Tuberculosis and Malaria (the Global Fund) and Gavi, the Vaccine Alliance, challenges related to human resource deficits can be detrimental to the impact of funding activities. In response, more international development agencies have provided funds toward broader health system initiatives and health workforce activities in particular [5, 6].

International funding for health workforce-related activities may broadly fall into one of two groups. Indirect funding for human resources activities such as per-diems and salary complements that are typically included as part of “running costs” of health projects or direct funding towards human resources for health through projects whose objective is to invest in human resources through activities such as training and policy development. The former is ubiquitous in most health projects but estimates of the later, the amount of donor funding being targeted toward direct investment in human resources for health activities, are few. In this study, we estimate the aggregation of these, as both are intended to build investments for health.

In 2012, a review of funding through three major international agencies – Gavi, the World Bank, and the Global Fund – for human resource activities showed that the average annual value of human-resources-related projects funded by these institutions was $1 million (0.5–1.5 m), $0.8 million (0.5–1.1 m), and $2.7 million (2.0–3.4 m), respectively. These estimates were based on funded activities in approved projects or grants and not a retrospective review of project or grant spending on human-resources-related activities [7]. Another study conducted a deep dive analysis into the Global Fund’s investments in human resources for health related projects [8]. This study found that approximately $1.4 billion of Global Fund grant funding had been allocated to human resources for health related activities by 138 recipient countries. Other project reviews and reports in the grey literature provide details on the types of human resources for health related activities undertaken by the various donor agencies [9, 10]. These reports, however, contain very limited information on actual disbursements and cover only a few donors.

This study addresses this gap by characterizing the sources, disbursing agencies, recipients, and trends in donor funding targeted toward human resources for health activities. We track development assistance for human resources for health from 1990 through 2016. We distinguish between the originating source of funds, disbursing agencies, and final recipients of the resources. We also describe the types of human resources for health activities these funds supported and examine how the allocation of funds for human resources for health relates to the allocation of overall development assistance for health and to need as determined by the disease burden and availability of health workers.

## Methods

We track development assistance for human resources for health from 1990 through 2016 using methods developed by the Institute for Health Metrics and Evaluation for tracking development assistance for health and data from various international development agencies [11–14]. The Institute for Health Metrics and Evaluation defines development assistance for health as the financial and non-financial resources transferred by development agencies to low- and middle-income countries with the primary goal of maintaining and improving health. In this study, we define development assistance for human resources for health as the development assistance for health specifically targeted toward health-worker-related activities. These include activities such as health workforce planning, development of guidelines for health workforce management, pre-service and in-service training, postgraduate fellowship opportunities, additional staff recruitment, general support for work with health workers and construction of health worker training institutions.

### Data sources

We utilize data from the following sources to generate our estimates of development assistance for health. For funds flowing through bilateral agencies such as the United States President’s Emergency Plan for AIDS Relief (PEPFAR), the Department for International Development (DFID), Australian Aid, Canada’s International Development Agency, global health initiatives such as the Global Fund, and multilateral institutions such as UN agencies and the EU, we extract data from the Organisation for Economic Co-operation and Development’s Creditor Reporting System (CRS) database. For funds disbursed by the Asian Development Bank and the Inter-American Development Bank, we use data downloaded from online databases, while for the Bill & Melinda Gates Foundation, the World Bank, and the African Development Bank, we use project-level data received through correspondence. Lastly, we use data from the Foundation Center for disbursements channeled through US foundations [15–18]. A detailed description of the methodology used to generate the development assistance for health estimates has been previously published [19].

### Estimating development assistance for human resources for health

To estimate development assistance for human resources for health, keywords for human resources for health related activities were used to identify projects targeted toward human resource processes. Because health projects may address cross cutting issues such as human resource shortages or address more than one health focus area such as maternal and child health, we account for such multi-purpose projects by assigning fractions of the total disbursements to specified components of the projects. Detailed explanation of the assignment methodology has been published elsewhere [19]. We also estimate the administrative expenses associated with these projects using information on grants disbursements and operational expenses from financial statements [11]. To generate the administrative expenses estimate, we divide operational expense by the amount of grant disbursed. This proportion provides a measure of the overhead costs associated with program implementation. Additional file 1: Figure S1 disaggregates administrative and program expense. The detailed list of the keywords used to identify the human resources for health related projects is also provided in the annex. In addition to English, the keywords are translated into and searched in eight other languages – French, Spanish, Swedish, German, Norwegian, Portuguese, Italian, and Dutch.

We divide the human-resources-related projects into six categories based on activities. These categories draw from existing human resources for health strengthening frameworks [20]. The categories are (i) Training, (ii) Policy and administrative management, (iii) Education, (iv) Staffing, (v) Infrastructure, and (vi) Other. Training activities captures pre-service and in-service training activities such as internships, seminars or on-the-job training. Policy and administrative management activities are those that serve to build leadership and management skills as well as those that focus on policy development. Education characterizes projects under which health workers are provided opportunities or sponsorship to pursue pre-service or postgraduate education. Staffing captures activities such as the hiring of consultants or additional specialists and personnel to increase the available labor supply. Infrastructure characterizes project activities such as the building of health training facilities or the provision of equipment to such health training facilities. All remaining project activities that are not classified in any of the preceding five categories are captured in “other”.

We generate estimates of development assistance for health targeted to human resources for health from 1990 through 2016. The estimates are reported in 2017 US dollars. Stata 13 was used for the analysis. For disbursing agencies such as the Global Fund and UN agencies for which data reported in the CRS database in earlier years are incomplete, we scale up annual disbursements to the levels estimated in IHME’s 2017 *Financing Global Health* report [11]. We do this by calculating the fraction of the total development assistance for health for these channels that is for human resources for health and multiplying this fraction from the CRS with the annual envelope estimate from IHME’s 2017 *Financing Global Health* report.

## Results

**Fig. 1:**
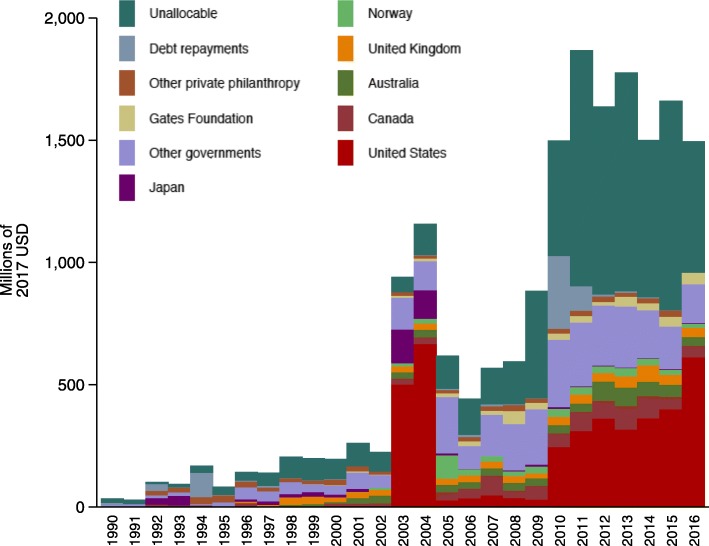
Development assistance for human resources for health by source, 1990–2016. Notes: “Other governments” includes Austria, Belgium, Denmark, Finland, France, Germany, Greece, Ireland, Italy, Luxembourg, Netherlands, New Zealand, Poland, Portugal, South Korea, Spain, Sweden, Switzerland, and United Arab Emirates. Health assistance for which we have no source information is designated as “Unallocable”. Sources: Authors’ analysis of data from the Institute for Health Metrics and Evaluation 2017 development assistance for health database

From 1990 through 2016, a total of $18.5 billion has been disbursed as development assistance to support human resources activities around the globe. In 2016, development assistance for human resources for health totaled $1.5 billion, 4.0% of total development assistance for health in that year. This total amount was a 44-fold increase in the amount of funding dedicated to human resources in 1990. Between 1990 and 2016, development assistance for human resources for health increased by 15.7% each year. Despite the positive annualized growth rate, over time this growth has not been consistent with intermittent periods of decline in resources targeted to human-resources-related activities. As a share of total development assistance for health, human resources for health has been under 7% over the entire study period. At its peak in 2004, it was at 6.2% of total development assistance. As a share of their total development assistance for health, South Korea, Australia, Canada, and Belgium devote the largest amount to health-worker-related activities. Figure 1 presents the annual total amount of development assistance targeted to human resources for health related activities by source country from 1990 through 2016. Until 2002, the annual disbursements for human-resources-related activities were negligible. Beginning in 2003, disbursements to health-related human resource activities are substantial. The US and Japan contributed the majority of funds in 2003 and 2004.

**Fig. 2:**
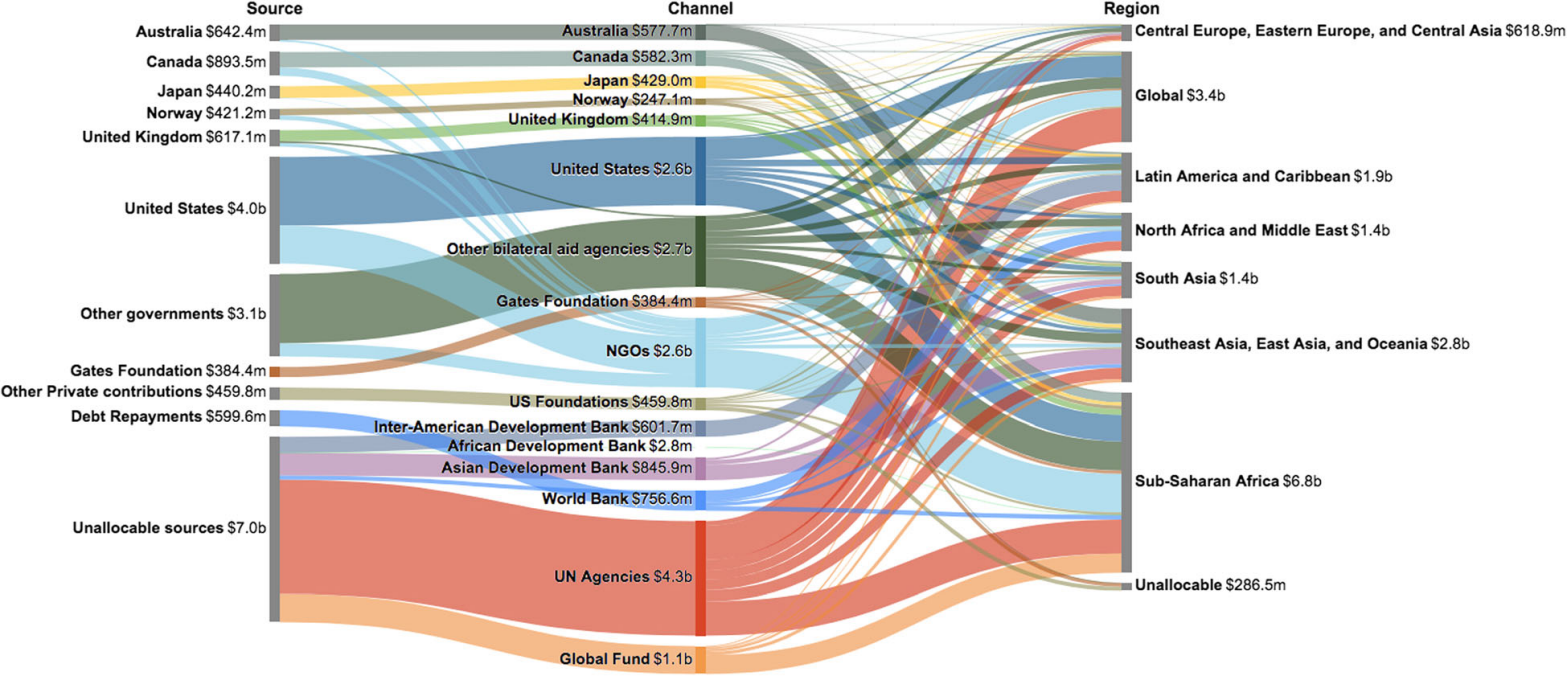
Flow of development assistance for human resources for health from source through disbursing channel to recipient region, 1990–2016. Notes: Values are given in 2017 US dollars. Global Burden of Disease super-regions are seven regions which group sub-regions based on cause of death patterns, as defined by the Global Burden of Disease Study 2016. “Other governments” includes Austria, Belgium, Denmark, Finland, France, Germany, Greece, Ireland, Italy, Luxembourg, Netherlands, New Zealand, Poland, Portugal, South Korea, Spain, Sweden, Switzerland, and United Arab Emirates. “Other bilateral aid agencies” includes Austria, Belgium, Denmark, Finland, France, Germany, Greece, Ireland, Italy, Luxembourg, Netherlands, New Zealand, Poland, Portugal, South Korea, Spain, Sweden, Switzerland, United Arab Emirates, and the European Commission. “UN Agencies” includes the Joint United Nations Programme on HIV and AIDS, United Nations Population Fund, United Nations Children’s Fund, and World Health Organization. Three regional types of CRS project-level data that did not perfectly align with GBD super-regions were allocated as follows: “Africa, regional” was allocated to “Sub-Saharan Africa” GBD super-region; “Asia, regional” was allocated to “Southeast Asia, East Asia, and Oceania” GBD super-region; “South & Central Asia, regional” was allocated to “South Asia” GBD super-region. Health assistance for which we have no source information is designated as “Unallocable sources”. Health assistance for which no recipient country or recipient region information is available is designated as “Unallocable”. Global initiatives are categorized as activities that are not confined to a specific region, and include health system strengthening and human resources for health. Sources: Authors’ analysis of data from the Institute for Health Metrics and Evaluation 2017 development assistance for health database

Figure 2 traces the flow of development assistance for human resources for health from the source country through the agency responsible for disbursing the funds to the region of the recipient organization from 1990 through 2016. Over this period, the main countries supporting human resources for health related activities were the US, Canada, Australia, and the UK. Each country contributed more than $500 million toward human resources for health related activities. The majority of these resources were disbursed through UN agencies, NGOs, US bilateral agencies such as PEPFAR, the Global Fund, the Asian Development Bank, and the World Bank. Countries in sub-Saharan Africa and Southeast Asia, South Asia, and Oceania received the bulk of these funds.

**Fig. 3:**
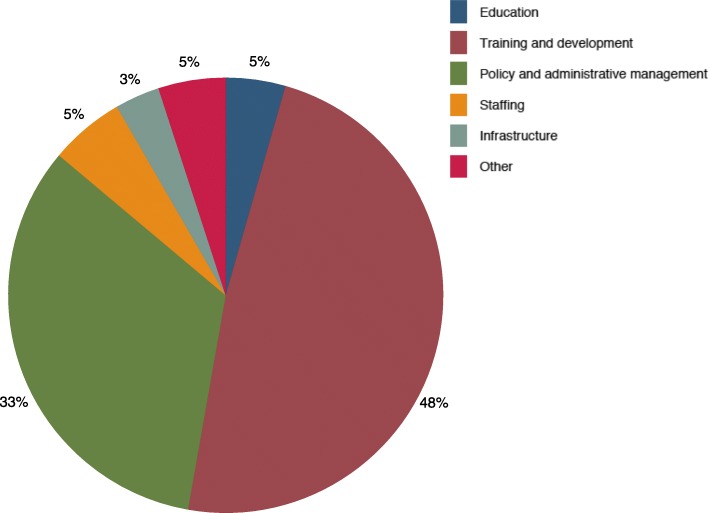
Development assistance for human resources for health by type of activity, 2014–2016. Sources: Authors’ analysis of data from the Institute for Health Metrics and Evaluation 2017 development assistance for health database

Figure 3 shows the types of human resource activities that have received support. The categories highlighted are training and personnel development, education, staffing, infrastructure, and policy and administrative management. The ‘Other’ category captures activities related to general support for work with health professionals as well as activities with too general descriptions to be classified into any of the other activity types. These include unspecified activities that facilitate collaboration between different types of health workers (for example, reproductive health and health reform specialists), improve job satisfaction, field test materials, travel costs for international conferences and meeting convening. From 2014 through 2016, training and personnel development was the most commonly supported investment in human resources for health related activity, while policy and administrative management, staffing, education, and infrastructure made up 33.3%, 5.5%, 4.6%, and 3.4%, respectively.

**Fig. 4:**
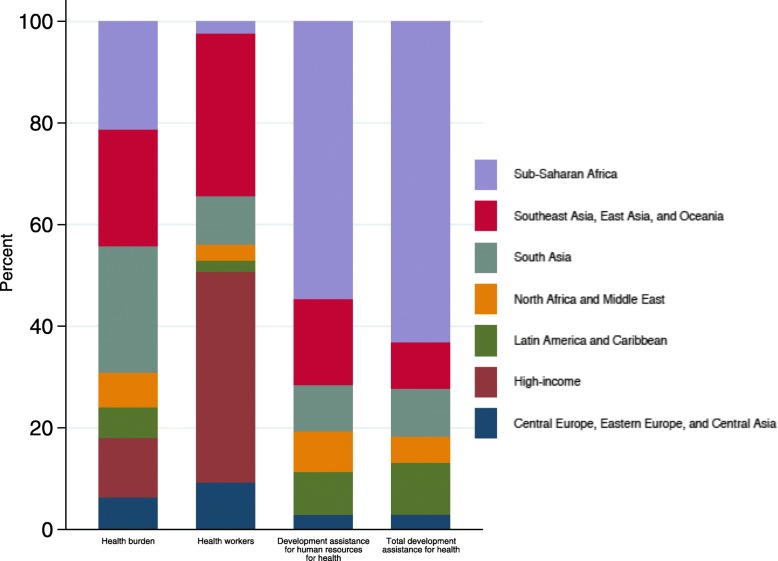
Health burden, health worker availability, and development assistance by global burden of disease region, 2014–2016. Notes: Global Burden of Disease super-regions are seven regions which group sub-regions based on cause of death patterns, as defined by the Global Burden of Disease Study 2016. Sources: Authors’ analysis of data from the Institute for Health Metrics and Evaluation 2017 development assistance for health database. Health burden information was obtained from Global Burden of Disease Study 2016 (citation: GBD 2016 DALYs and HALE Collaborators. Global, regional, and national disability-adjusted life years (DALYs) for 333 diseases and injuries and healthy life expectancy (HALE) for 195 countries and territories, 1990–2016: a systematic analysis for the Global Burden of Disease Study 2016. *The Lancet*. 14 Sept 2017: 390;1260–344.). Health workers information was obtained from World Health Organization Global Health Observatory data repository (citation: http://apps.who.int/gho/data/node.main.A1443?lang=en&showonly=HWF)

Figure 4 highlights the distribution of disease burden, availability of health workers, and development assistance by global burden of disease super-region. The burden of disease is highest in three regions of the world – sub-Saharan Africa; Southeast Asia, East Asia, and Oceania; and South Asia. The majority of development assistance for health and human resources for health assistance goes to sub-Saharan Africa. Health worker availability is highest in the high-income super-region and Southeast Asia, East Asia, and Oceania and smallest in sub-Saharan Africa and Latin America and Caribbean.

## Discussion

This study tracks development assistance for human resources for health using data from the main international development agencies and bilateral donors. We find that from 1990 through 2016, financial resources committed toward human resources for health related activities has increased 44- fold, from $33.9 million to $1.5 billion. The number of donors supporting human resources activities has also increased, as have the numbers of disbursing agencies and types of activities funded.

Of note is the striking similarity between the growth in resources targeted toward human resources activities and the evolution of initiatives on the global health landscape. The establishment of global health initiatives such as the Global Fund, Gavi, and PEPFAR seems to have ushered in a period of greater concentration and commitment of resources toward human resources for health. In particular, the amounts of resources targeted to human-resources-related activities quadrupled from 2002 with the establishment of PEPFAR in 2003. PEPFAR is a US government initiative established to provide an aggressive and bold response to the HIV/AIDS epidemic [21]. In addition to accelerating the provision of antiretroviral drug treatment in high-burden HIV countries, it has also attempted to address broader health system issues such as health worker shortages. Additionally, even more disease-specific entities such as the Global Fund have acknowledged the challenges related to human resources constraints in low- and middle-income countries by allowing countries to apply for funding in support of human-resources-related activities [7]. Gavi also responded to this constraint through the establishment of a health system funding stream that allowed countries to access funds for broader health system issues like shortages and poor distribution of human resources for health [5].

As the largest source of funding for human resources for health related activities since 2003, the US has channeled the majority of its funds through its bilateral agency PEPFAR and non-governmental entities. In particular, the re-signing of that project in 2008 included a goal of training and retaining 140,000 more health workers across PEPFAR focus countries [7]. For most other sources of human resources for health funding, their own bilateral agencies have been the main disbursing agencies of these funds. Although other agencies have supported human resources activities that have broader utility in the health system, because the bulk of funding flowed through disbursing agencies with specific disease focus, the type of human-resources-related activities funded and the target regions have reflected this orientation. In this regard, sub-Saharan African countries in which the HIV/AIDS epidemic was catastrophic attracted and secured much of the funding related to human resources for health. The challenge here is that in health systems where there were shortages in health workers, redirecting health worker efforts to particular disease areas has been criticized as being detrimental to the broader health system [22–24].

The specific human resources for health activities undertaken seem to have been shaped by the goals of the funding entities. The majority of the investment in human resources for health activities funded are related to training and policy and administrative management. These specifically refer to in-service training and assistance related to developing the appropriate policies, guidelines, and assessment for the management of human resources for health. Grant requirements, project eligibility guidelines, and project cycle timelines of international development entities have been highlighted as a constraint to undertaking long-term projects that can address some of the systemic challenges of the human resources for health problem [7, 9]. Implementers need to show results to funders in the medium-to-short term, and that has driven the short-term nature of the activities that have been undertaken. Short-term project “running costs” activities seem to have consumed the majority of the assistance for human resources related activities while the smallest share of resources has been put toward activities that will help resolve the systemic challenges in the health labor market in the recipient countries. In particular, infrastructure and education receive only 3% and 5% of resources, respectively. Education captures activities such as sponsorship for completion of pre-service or post-service degrees, and infrastructure is related to the construction of more health training facilities. These two activities are the most directly related to activities that can fundamentally increase the stock of health workers. Nonetheless, these are the ones for which funding is limited. One sustainable way in which staffing challenges have been addressed in some instances is by donors providing salary top-ups and other pay incentives. For instance, in Malawi, the UK’s Department for International Development and the Global Fund’s initiative of collaborating with the government to provide emergency support for health worker recruitment has been held up as an example of sustainable solutions to a major health system challenge in a resource-scarce environment [25].

The alignment between health burden, health worker availability, and funding is mixed. Relative to its disease burden, South Asia has modest health worker availability and receives a small share of both development assistance for health overall and specifically for human-resources-related activities. Southeast Asia, East Asia, and Oceania receives a relatively smaller share of overall development assistance for health, whereas the share of development assistance for human resources for health is larger. Besides Latin America and Caribbean, sub-Saharan Africa shows the smallest share of global health workforce availability and receives the majority of development assistance for human resources for health. The share of human resources for health related assistance targeted to Southeast Asia, East Asia, and Oceania is more aligned to its disease burden. Nonetheless, compared to the overall development assistance for health its share is disproportionately small. This misalignment speaks to a larger discourse in the global health spending arena regarding the criteria used by development agencies for the allocation of resources. This is an issue that is also important for the attainment of the Sustainable Development Goals. Several studies exist that suggest that the allocation of aid is largely driven by donors’ own strategic interests [26–28]. In other words, the amount of funding received by a recipient country is primarily a result of donor country objectives and not recipient country need. Some have argued that such a criteria limits the effectiveness of aid resources and makes recipient countries worse off in the long run. Without adequate resources targeted toward the areas most in need, improvements in health and well-being will be modest.

Furthermore, the out migration of health workers presents a challenge to efforts to increase the stock of human resources for health available in low and middle income countries. The “pull” and “push” factors fueling migration have been extensively documented in the literature [1, 29–35]. In 2010, the 193 member states of the WHO adopted the WHO Global Code of Practice on the International Recruitment of Health Personnel in recognition of the challenges associated with the ethical recruitment of health professionals globally [36]. One of the articles in the code encouraged high-income countries to provide financial and technical assistance to low-income countries to mitigate the impact of health personnel emigration. Future work will examine the association between the transfer of these resources and out migration of health workers.

This study has several limitations. First, although our dataset comprehensively captures the major donors that report to the Organisation for Economic Co-operation and Development’s Development Assistance Committee, it does not include development assistance for health contributions from emerging donors like China, Brazil, India, and Russia or south-south cooperation initiatives due to lack of data availability. It therefore excludes China’s contribution to development assistance for human resources for health through the deployment of medical teams to support and train health workers in other low- and middle-income countries. Also, our database does not include project description at a level for which we could extract development assistance for human resources for health estimates for the UN agencies and Global Fund. We therefore supplemented our data with project-level data from the CRS. However, because CRS data are underreported for earlier years, we rescale our estimates using the envelope from our database in order to get more accurate estimates for those agencies for which we use CRS data as supplement. The identification of the relevant human resources for health related projects was also done using keywords included in the project descriptions; therefore, projects for which human resources activities were undertaken but not mentioned in the project description will not be captured in our estimates. Lastly, our analysis does not include development assistance for health channeled through Gavi. This is because the descriptions of Gavi projects provided in all available public databases did not provide the level of detail needed to ascertain whether these disbursed resources were related to human resources for health.

## Conclusion

As of 2016, less than 4% of development assistance for health could be tied to funding for human resources. Given the central role skilled health workers play in health systems, in order to make credible progress in reducing disparities in health and attaining the goal of universal health coverage for all by 2030, it may be appropriate for more resources to be mobilized in order to guarantee adequate manpower to deliver key health interventions.

## Additional file

Additional file 1: Annex. (PDF 489 kb)
