# Supplementary material for: Donor financing of human resources for health, 1990–2016: an examination of trends, sources of funds, and recipients
Source: Global Health. 2018 Oct 17;14:98. doi: 10.1186/s12992-018-0416-z (PMC6192106; doi:10.1186/s12992-018-0416-z)
Supplement: Supplementary file 1 — Annex. (PDF 489 kb) [file 12992_2018_416_MOESM1_ESM.pdf]

ANNEX

Keywords used to identify HRH related projects

global hrh_level " INFRASTRUCTUR" " MEDICAL EQUIPMENT" " SURGICAL EQUIPMENT" " HOSPITAL EQUIPMENT" " HOSPITAL EQMT " " BUILDINGS " " HEALTH FACILIT" " CONSTRUCT" " MEDICAL SCHOOL" "CENTERS OF EXCELLENCE" " TRAINING " " CAPACITY " " SKILLED WORKER" " HEALTH WORKER" " SKILLED STAFF " " HEALTH PROFESSIONAL" " HUMAN RESOURCE" " HUMAN CAPITAL " " IMPROVED CAPACITIES " " MEDICAL WORKER" " HEALTH CARE PERSONNEL " " WORKFORCE " " ADMINISTRATIVE " " MEDICAL EDUCATION " " HEALTH EDUCATION " " CONTINUING EDUCATION " " HEALTH MANAGEMENT" " MANAGEMENT AND COORDINATION " " ADMINISTRATIVE MANAGEMENT " " MANAGEMENT AND ADMINISTRATION " " STRENGTHENING INSTITUTIONAL CAPACIT" "NURSE" "DOCTOR" "PHYSICIAN" "MIDWIFE" "MIDWIVES" "MEDICAL LABORATORY SCIENTIST" "SURGEON" "SPECIALIST" "PHARMACIST" "COMMUNITY HEALTH WORKER" "EMPLOYMENT" "HEALTH LABOR" "LABOR MARKET" "PERSONNEL" "MEDICAL PRACTIONER" "DENTAL PRACTIONER" "TASK SHIFTING"

Program areas

global train_level "TRAINING" " TRAIN " "TRNG" "CAPACITY" "CAPACITIES" "CAPACITE" "ENTRAINEMENT" "LEARNING" "INTERNSHIP" " FORMATION" "WORKSHOP" "COURSES"

global educ_level " EDUC" "SCHOLARSHIP" "BOURSES"

global mgmt_level " ADMIN" "MANAGEMENT" "LEADERSHIP" "POLICY" "POLICIES"

global pers_level "PERSONNEL DEVELOPMENT" "DVPT" "DEVPT"

global staff_level "FELLOW" "SALAR" "VOLUNTEER" "STAFFING" "PROVISION OF SURGICAL TEAM" "CONSULTANCY" "SECONDMENT" "WORK PERMIT" "TA SUPPORT" "TECHNICAL ASSISTANCE"

global budget_level "GENERAL BUDGET SUPPORT"

global infra_level " INFRASTRUCTUR" " MEDICAL EQUIPMENT" " SURGICAL EQUIPMENT" " HOSPITAL EQUIPMENT" " HOSPITAL EQMT " " BUILDINGS " " HEALTH FACILIT" " CONSTRUCT" " MEDICAL SCHOOL" "CENTERS OF EXCELLENCE"

Figure S1: Development assistance for human resources for health by type of expense, 1990-2016

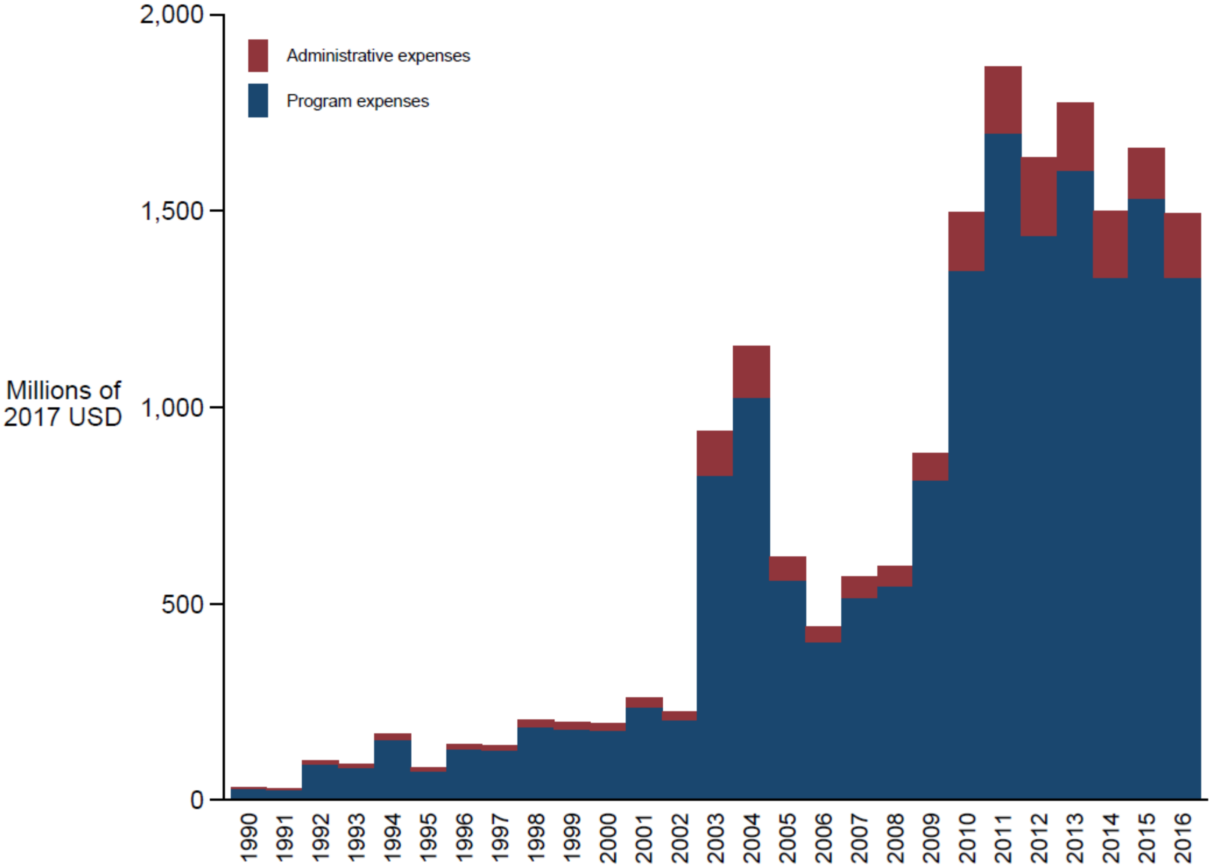

Note: Administrative expenses is calculated by dividing operational expense by the amount of grant disbursed.

Sources: Authors' analysis of data from the Institute for Health Metrics and Evaluation 2017 development assistance for health database.
